# Recent Progress in Electrochemiluminescence of Halide Perovskites

**DOI:** 10.3389/fchem.2021.629830

**Published:** 2021-03-19

**Authors:** Yue Cao, Jun-Jie Zhu

**Affiliations:** State Key Laboratory of Analytical Chemistry for Life Science, School of Chemistry and Chemical Engineering, Nanjing University, Nanjing, China

**Keywords:** electrochemiluminescence, stability, biosensing, interface manipulation, halide perovskite

## Abstract

Halide perovskites are a rapidly developing class of solution-processable semiconductors which, to date, have a huge impact across several scientific communities. The remarkable photophysical attributes of halide perovskites illustrate their considerable potential in the electrochemiluminescence (ECL) realm. Over the past 4 years, great progress has been achieved in using halide perovskites as ECL emitters. In this mini-review, the basic characteristics, synthetic approaches, and ECL mechanisms for halide perovskite emitters are first introduced. To the best of our knowledge, most of the reported ECL-active halide perovskites and their disclosed unique features are detailly summarized. Stabilization and interface manipulation strategies for desirable ECL performance are further highlighted. The preliminary halide perovskites-related ECL applications are finally discussed, and prospects are also anticipated.

## Introduction

Electrochemiluminescence (ECL) is a light-emitting phenomenon arisen from electrochemical reactions between electrogenerated species in the vicinity of electrode. Known for excellence in low background, high sensitivity, and simple instrument, ECL has been acknowledged as a versatile analytical technology in life analysis, environmental monitoring, and pharmaceutical research, etc. (Ma et al., 2020). The emitters play a crucial role as electronic-to-optical transducers in ECL systems, whose exploitation and utilization are always the direction of efforts. Since the pioneering investigation on the ECL of silicon nanocrystals (NCs) (Ding et al., 2002), various ECL available semiconducting NCs have sprung up exuberantly, featuring a solution-processable colloidal state with quantum size effect, malleable surface chemistry, favorable optical property, and stable chemical composition (Miao, 2008).

The recent surge of interest in halide perovskites (PeNCs) has emerged in light-emitting diodes, lasers, and solar cells because of their appealing optoelectronic properties (Stoumpos and Kanatzidis, 2016). In 2016, Huang et al. first observed the ECL phenomenon during the study of all-inorganic CsPbBr_3_ PeNCs, which opens the doors to the fundamental ECL research and application of PeNCs. After nearly 4 years of development, although various ECL-active PeNCs and a few unique merits have been disclosed, ECL research on PeNCs is still in its infancy and the subject of heightened concern. A recent review majors in the ECL performance of various PeNCs systems in either organic or aqueous media (Kong et al., 2020). In this mini-review, therefore, we present a detailed summary of the progress in using PeNCs as ECL emitters, which sketches the creative trajectory from the basic investigation of ECL-active PeNCs to the design strategies for desirable ECL property and resulting applications, hoping to provide readers with a comprehensive understanding of relevant contents and new ideas.

## Basic Characteristics and Synthetic Approaches

PeNCs are characterized by the general formula AMX_3_, where A is a monovalent cation [e.g., Cs^+^, CH_3_NH_3_^+^ (MA^+^), HC(NH_2_)_2_^+^ (FA^+^)], M is a bivalent metal cation (e.g., Pb^2+^, Ge^2+^, Sn^2+^, etc.), and X is a halide anion (Cl^−^, Br^−^, I^−^). Octahedral MX_6_^4−^ is formed by the coordination of M and X at its center and vertex, respectively. The crystal structure of PeNCs consists of anionic 3D networks of corner-sharing MX_6_^2−^ octahedra and the cavities occupied by A.

ECL emitters of PeNCs are mainly solution-processed colloidal NCs or quantum dots (QDs) via a hot-injection route (HIR) or a ligand-assisted reprecipitation method (LARM). Take the case of CsPbX_3_ NCs formation, HIR is conducted by swiftly injecting cesium oleate into an octadecene (ODE) solution containing PbX_2_, oleylamine (OAm), and oleic acid (OA) at high temperature (>140^o^C) in a nitrogen atmosphere (Xue et al., 2017). In contrast, LARM is performed under mild reaction conditions without heating and nitrogen protection, which begins by dissolving precursors (CsX and PbX_2_) in a “good” solvent (e.g., N, N-dimethylformamide, dimethylsulfoxide), and then transferring into a “poor” antisolvent (typically toluene) to reprecipitate NCs in the presence of ligands (Cao et al., 2020b). Besides, microwave was also utilized for one-step synthesis of CsPbX_3_ NCs as ECL emitters via directly irradiating the ODE solution containing precursors (Cs_2_CO_3_ and PbX_2_) and ligands (OA and OAm) (Wang et al., 2020c).

The ECL mechanism of PeNCs is generally investigated following both annihilation and coreactant routes, which is basically consistent with the previously proposed nanomaterial ECL systems (Miao, 2008). The annihilation route transmits the ECL signal from single emitters via the direct transferring of exergonic electron between the electrogenerated reduced and oxidized radicals of PeNCs, which can be described as follows (Reactions 1–4):

$$\text{PeNCs}-\text{e }\to {\text{ PeNCs}}^{+•}$$ (1)

$$\text{PeNCs}+\text{e }\to {\text{ PeNCs}}^{-•}$$ (2)

$${\text{PeNCs}}^{+•}{\;+\text{ PeNCs}}^{-•}\;\to {\text{ PeNCs}}^{\text{∗}}{\;+\text{ PeNCs}}^{\text{0}}$$ (3)

$${\text{PeNCs}}^{\text{∗}}\to \text{ PeNCs }+\text{ hv}$$ (4)

The coreaction mechanism requires an appropriate coreactant to assist emitters for the ECL signal. Taking the example of tripropylamine (TPrA), a typical “oxidation-reduction” coreactant for PeQDs, the coreactant route can be described as the following Reactions 5–8. Briefly, TPrA undergoes electro-oxidization and deprotonation to form a highly reductive radical intermediate TPrA^•^. Meanwhile, PeNCs are electro-oxidized to PeNCs^+•^. The radiative recombination of TPrA^•^ and PeNCs^+•^ produces excited PeNCs^∗^ for ECL emission. Searching for promising coreactants is beneficial to expand potential ECL applications of PeNCs. As far as we know, PeNCs mainly emit anodic ECL with the coreactants including TPrA (Cai et al., 2018), 2-(dibutylamino)ethanol (DBAE) (Li et al., 2019), ascorbic acid (AA) (Cao et al., 2020c), ethyl acetate (EA) (Xue et al., 2017), and hydrogen peroxide (H_2_O_2_) (Huang et al., 2017). Besides, the cathodic ECL of certain PeNCs was also observed in the presence of K_2_S_2_O_8_ (Peng et al., 2020) or benzoyl peroxide (BPO) (Cao et al., 2019).

$$\text{TPrA}-\text{e }\to {\text{ TPrA}}^{•}{\;+\text{ H}}^{+}$$ (5)

$$\text{PeNCs}-\text{e }\to {\text{ PeNCs}}^{+•}$$ (6)

$${\text{PeNCs}}^{+•}{\;+\text{ TPrA}}^{•}\;\to {\text{ PeNCs}}^{\text{∗}}\;+\text{ Products}$$ (7)

$${\text{PeNCs}}^{\text{∗}}\to \text{ PeNCs }+\text{ hv}$$ (8)

## Available Halide Perovskite Emitters

Multifarious ECL-active PeNCs spring up vigorously, whose fundamental studies and practical uses have set off a new research upsurge. To the best of our knowledge, the main ECL parameters for the reported PeNCs-based systems are summarized in Table 1, and several representative ones are described in detail here.

**TABLE 1:** Main basic and ECL parameters for various PeNCs-based systems.

PeNCs	Morphol.	Size nm	λ_PL_ ^a^ nm	Coreactant	λ_ECL_ ^b^ nm	fwhm_ECL_ nm	ϕ_ECL_ ^c^ %	Ref.
CsPbBr_3_	cube	12–15	515	TPrA/BPO	519	20	—	Huang et al. (2016)
CsPbBr_3_	cube	∼10–25	516	H_2_O_2_	518	20	—	Huang et al. (2017)
CsPbBr_3_	cube	8	509	EA	515	24	500	Xue et al. (2017)
CsPbBr_3_	cube	∼20	518	TPrA	∼560	∼41	—	Cai et al. (2018)
CsPbBr_3_	cube	∼8	500	S_2_O_8_^2−^	570	∼62	1.6	Peng et al. (2020)
CsPbBr_3_	cube	9	519	EA	—	—	—	Hao et al. (2019)
CsPbBr_3_	cube	9.57	509	AA	506	∼100	—	Wang et al. (2020b)
CsPbBr_3_	cube	13	511	TPrA	569	26	—	Qiu et al. (2019)
CsPbBr_3_	cube	∼20	520	H_2_O_2_/TPrA	—	—	—	Wang et al. (2020c)
MAPbBr_3_	nanowire	500 × 50	531	TPrA/S_2_O_8_^2−^	535	25	—	Tan et al. (2017)
FAPbBr_3_	cube	13 ± 2	529	TPrA	534	31.3	—	Wang et al. (2020a)
Cs_3_Bi_2_Br_9_	dot	4.8 ± 1.24	389	TPrA/BPO	—	—	—	Cao et al. (2019)
MAPbCl_1.08_Br_1.92_	dot	3.5 ± 0.15	450	TPrA	473, 744	∼41, ∼200	—	Wusimanjiang et al. (2019)
Rb_0.2_Cs_0.8_PbBr_3_	cube	10 ± 2	514	DBAE	510	20	—	Chen et al. (2020)
Sb^3+^-CsPbBr_3_	cube	21	520	TPrA	520	19	—	Jia et al. (2019)
Ce^4+^-CsPbBr_3_	dot	5	468	TPrA	522	26	—	Fu et al. (2020b)
CsPbBr_3_-CeO_2_	column	70 × 12	520					
CsPbBr_3_-TOP	cube	∼13	523	OAm, AA	523	∼30	57.08	Cao et al. (2020b)
CsPbBr_3_-Ag_2_S	cube	8–10	515	TPrA	∼520	∼50		Fu et al. (2020a)
CsPbBr_3_-DBAE@SiO_2_	cube^d^	7.6^d^	518	DBAE	∼530	∼47	410	Li et al. (2019)
CsPbBr_3_@silica gel	cube^d^	19^d^	516	DBAE	517	39	—	Li et al. (2020)
CsPbBr_3_@HCNS	—	—	507	AA	525.7	34.5	—	Cao et al. (2020c)
CsPbBr_3_-NCDs@HZIF-8	cube^d^	∼4.0^d^	504	NCDs	539	41.3	—	Cao et al. (2020a)

^a,b^ λ_PL_ and λ_ECL_ represent the maximum emission wavelength of PL and ECL, respectively.

^c^ φ_ECL_ represents ECL efficiency relative to the standard Ru(bpy)_3_^2+^/TPrA system.

^d^ the actual morphology and size of CsPbBr_3_ in hybrids.

**FIGURE 1:**
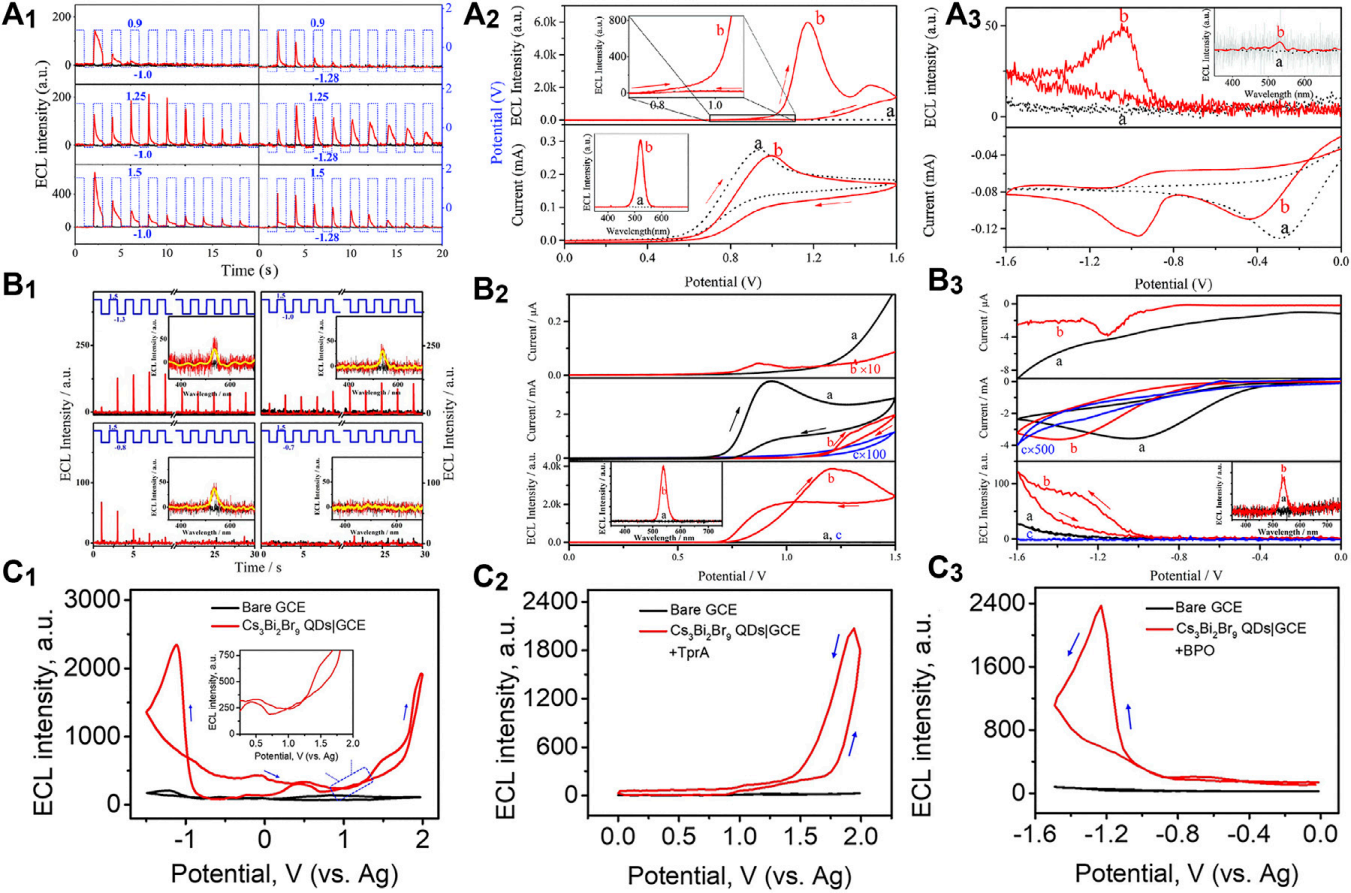
**(A**_**1**_ **)** Oxidation initiated transient electrochemiluminescence (ECL), **(A**_**2**_ **)** anodic coreactant ECL (10 mA TPrA), and **(A**_**3**_ **)** cathodic coreactant ECL (5 mM BPO) of CsPbBr_3_ NCs|GCE in air-free dichloromethane containing 0.10 M tetra-*n*-butylammonium hexafluorophosphate (TBAPF_6_). Reproduced with permission from Huang et al. (2016). Copyright (2016) The Royal Society of Chemistry. **(B_1_)** Oxidation initiated transient ECL, **(B**_**2**_ **)** anodic coreactant ECL (10 mA TPrA), and **(B**_**3**_ **)** cathodic coreactant ECL [100 mM (NH_4_)_2_S_2_O_8_] of MAPbBr_3_ NCs|GCE in air-free 0.10 M PBS. Reproduced with permission from Tan et al. (2017). Copyright (2017) American Chemical Society. **(C**_**1**_ **)** Annihilation ECL, **(C**_**2**_ **)** anodic coreactant ECL (10 mM TPrA), and **(C**_**3**_ **)** cathodic coreactant ECL (10 mM BPO) of Cs_3_Bi_2_Br_9_ QDs|GCE in a binary organic solution of acetonitrile and toluene containing 0.05 M TBAPF_6._ Reproduced with permission from Cao et al. (2019). Copyright (2019) American Chemical Society.

Fully-inorganic CsPbBr_3_ NCs are the most reported PeNCs as ECL emitters. Huang et al. (2016) first investigated the ECL behaviors of cubic CsPbBr_3_ NCs (12–15 nm). The direct charge injection produced various charged NCs, which further generated charge-transfer-mediated ECL in an annihilation or coreaction pathway (Figure 1A). The ECL spectrum of the CsPbBr_3_ NCs displayed a sole and symmetric peak centered at 519 nm, and the full width at half maximum (fwhm) was only about 20 nm, which is much narrower than classical ECL luminophores, that is, for example, commercial Ru(bpy)_3_^2+^ over 100 nm. Transient ECL signal occurred via the electrochemical oxidation of negative-charged NCs, and no ECL signal was obtained conversely, indicating the electrochemically switchable ECL property (Figure 1A_1_).

Organometallic PeNCs with A as organic cations have attracted board interest in optoelectronic and photovoltaic devices. By using ECL technology, the redox and charge transfer natures of highly crystalline MAPbBr_3_ nanowires (500 × 50 nm) were first studied in phosphate buffer solution (PBS) by Tan et al. (2017). MAPbBr_3_ NCs could be electrochemically oxidized or reduced to several opposite charged states. The redox nature regulated the charge transfer process for ECL emission via an annihilation route or a coreaction route with TPrA or (NH_4_)_2_S_2_O_8_ as the coreactants (Figure 1B). Electrochemically switchable ECL was also observed, but transient ECL was only obtained via injecting electrons into the positive-charged NCs (Figure 1B_1_). Besides, their anodic ECL spectrum demonstrated a single and symmetric peak around 535 nm with high color purity (fwhm, 25 nm), which was almost identical to the photoluminescence (PL) one. Soon after, anodic ECL of FAPbBr_3_ NCs (13 ± 2 nm) centered at 534 nm with an fwhm of 31 nm was also discovered by injecting holes into the negative-charged NCs or using the TPrA coreactant (Wang et al., 2020a).

Lead-based PeNCs are currently the best performing ECL emitters; however, the toxicity of lead hinders the commercial prospects. In theory, lead can be replaced with other low-toxic metals. Through an all-round improvement of LARM, Cao et al. (2019) synthesized lead-free Cs_3_Bi_2_Br_9_ QDs with good monodispersity, remarkable stability, and highly passivated surface, thereby affording unprecedented optical property with a PL quantum yield up to 37%. The high-quality QDs were first discovered with both anodic and cathodic ECL emission in either an annihilation or a coreaction route (Figure 1C), which might open an avenue for the design of lead-free PeNCs as eco-friendly and stable ECL emitters.

Halogen anion adjustable is one of the outstanding characters of PeNCs, which can lead to bright PL emission over the entire visible spectral region. Wusimanjiang et al. (2019) synthesized mixed-halogen perovskite of MAPbCl_1.08_Br_1.92_ QDs (3.5 nm). Rare sky-blue ECL emission centered at 473 nm was first achieved, demonstrating the feasibility of halogen anion-tunable ECL spectra. Besides, mixed-monovalent cations can also regulate the ECL properties of PeNCs. Chen et al. (2020) prepared Rb_x_Cs_1-x_PbBr_3_ NCs by partly replacing Cs^+^ with Rb^+^. With the increase of Rb^+^ content, the ECL spectra blue shifted gradually, and the ECL intensity displayed an up-and-down trend. Heterovalent substitution is also proposed to tailor the optoelectrical attributes of PeNCs. Jia et al. (2019) directly introduced Sb^3+^ (Sb/Pb = 1:3, molar ratio) in the synthesizing procedure of CsPbBr_3_ NCs via HIR. The partial replaces of Pb^2+^−Br bonds with strong Sb^3+^−Br bonds enlarged the band gap and preserved highly passivated surface states. Meanwhile, the doping behavior induced more vacancies and impurities, facilitating radiative charge transfer and electron injection/transfer efficiency for both enhanced PL and ECL. Also, Fu et al. (2020b) reported a similar Ce^4+^ doping process and a CeO_2_-conversion strategy, achieving modulation of electrochemical and radiative-charge-transfer behaviors of CsPbBr_3_ NCs for tunable PL and ECL. The facile and large-scale tunability of ECL property via ions adjustable might open novel probabilities to design multicolor emitters.

## Stabilization Strategy for Desirable ECL

The inherent vulnerability of PeNCs toward the external environment severely restricted their ECL progress. Although significant advances have been achieved in enhancing the stability of PeNCs by surface engineering or embedding into inert matrices, these protective layers are at a compromise with charge and mass transports, which inevitably lower the ECL efficiency. Thus, many strategies have been proposed for suitable constructions in the trade-off between ECL efficiency and structural stability.

**FIGURE 2:**
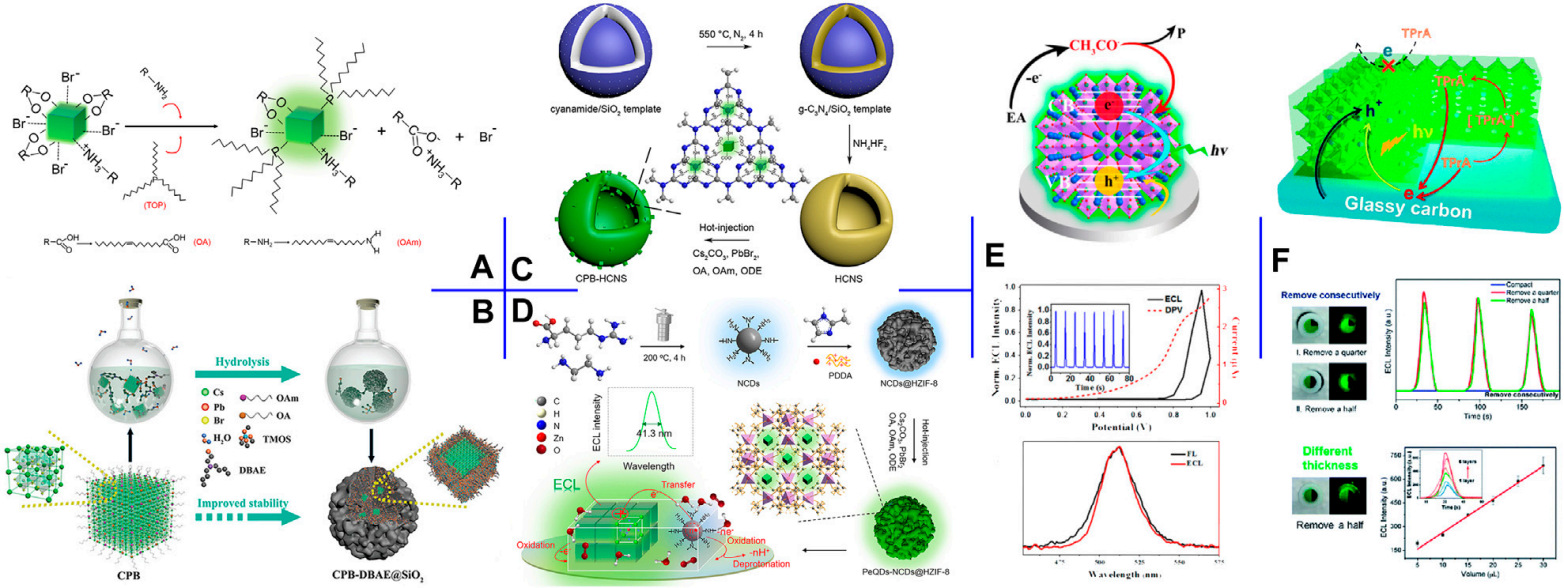
**(A)** Surface engineering process of CsPbBr_3_ NCs with OAm and TOP additives. Reproduced with permission from Cao et al. (2020b). Copyright (2020) The Royal Society of Chemistry. **(B)** Schematic illustration of the preparation process of CsPbBr_3_-DBAE@SiO_2_ ternary hybrids. Reproduced with permission from Li et al. (2019). Copyright (2019) Wiley-VCH. **(C)** Schematic illustration of the construction process of CsPbBr_3_-HCNS nanocomposite. Reproduced with permission from Cao et al. (2020c). Copyright (2020) American Chemical Society. **(D)** Schematic illustration of the construction process of CsPbBr_3_-NCDs@HZIF-8 nanocomposite. Reproduced with permission from Cao et al. (2020a). Copyright (2020) American Chemical Society. **(E)** ECL mechanism, the ECL-potential and DPV curves, and the PL and ECL spectra of the high-quality CsPbBr_3_ QDs film in EA. Reproduced with permission from Xue et al. (2017). Copyright (2017) American Chemical Society. **(F)** Schematic illustration of the three-phase heterostructure strategy and the corresponding ECL responses. Reproduced with permission from Qiu et al. (2019). Copyright (2019) The Royal Society of Chemistry.

ECL is particularly sensitive to surface chemistry because charges must pass through the surface before being injected into NCs to trigger the ECL (Ding et al., 2002). Thus, surface modification with robust ligands can form a highly passivated surface of PeNCs. Cao et al. (2020b) synthesized surface-passivated CsPbBr_3_ NCs by post-synthetic treatment with tri-n-octylphosphine (TOP) (Figure 2A). As a Lewis base and a highly branched ligand, TOP succeeded in reducing the NCs surface defects for enhanced ECL intensity and delaying the degradation of PeNCs. Passivating PeNCs surface with noble metal-based nanoparticles (NPs) can enhance the charge injection/transfer capacity while increasing stability. Fu et al. (2020a) introduced silver diethyldithiocarbamate into the CsPbBr_3_ NCs crude solution. During the process, Ag_2_S NPs were generated *in situ* on the CsPbBr_3_ NCs surface. The nano-heterostructure guaranteed enhanced aqueous stability and radiative charge transfer, thereby affording 9-fold ECL enhancement in an aqueous electrolyte.

Silica has been recognized as an inert and robust coating to enhance the stability of guests. Li et al. (2020) encapsulated CsPbBr_3_ NCs in silica gel via injecting CsBr aqueous solution into Cs_4_PbBr_6_ NCs and tetramethoxysilane (TMOS) hexane solution. The small amount of water triggered the conversion of Cs_4_PbBr_6_ NCs to CsPbBr_3_ NCs and the *in-situ* formation of waterproof silica gel. The abundant CsBr not only reduced the transformation rate but also greatly enhanced the conductivity of silica gel. Accordingly, the perovskite hybrids achieved strong ECL and still maintained their optical property after thirty-day storage in CsBr solution. To reduce the negative impact of silica coating on charge and mass transport, Li et al. (2019) co-encapsulated CsPbBr_3_ QDs and coreactant into silica matrix through in-situ hydrolysis of TMOS (Figure 2B). DBAE was selected as the optimal coreactant because its tertiary amine could act as both coreactant for CsPbBr_3_ QDs and catalyst for TMOS hydrolysis, and its hydroxyl group could be cross-condensed with TMOS and interact with silanol groups via hydrogen bonding. The perovskite-derived ternary hybrids achieved multifold ECL efficiencies (vs. Ru(bpy)_3_^2+^/TPrA) and preserved 55% of initial ECL intensity after 48 h storage in ambient condition with 100% relative humidity.

Semiconductor heterojunction can regulate the transfer and recombination of carriers in its hybrid structure. Cao et al. (2020c) prepared hollow g-C_3_N_4_ nanospheres (HCNS) *via* polycondensation of cyanamide in a silica template, and CsPbBr_3_ NCs further grew *in situ* within HCNS *via* HIR. In this work, HCNS as an ideal scaffold not only protected the internal CsPbBr_3_ NCs but also provided a matching band edge for upgraded ECL performance (Figure 2C).

Metal-organic frameworks (MOFs) are recognized as fulfilling nanocarriers, attributable to tunable porosity, high surface area, and structural diversity. Cao et al. (2020a) designed a ternary nanocomposite by successively loading aminated carbon dots (NCDs) and CsPbBr_3_ QDs *in situ* into the hierarchical zeolite imidazole framework-8 (HZIF-8) (Figure 2D). In this confined structure, HZIF-8 acted as a robust matrix for the loading of guest QDs and significantly enhanced the stability of CsPbBr_3_ QDs. Meanwhile, NCDs not only contained sufficient amines as efficient intra-nanomaterial coreactants of PeQDs for self-enhanced ECL but also improved the charge injection/transfer capacities of the hybrids. Consequently, the ternary architecture guaranteed high ECL performance in terms of stability and efficiency.

## Interface Investigation and Manipulation

The present ECL study of PeNCs mainly focuses on their solid-state film. Thus, the interfacial reaction and manipulation are of paramount importance for better ECL performance. Cai et al. (2018) deposited an optimized amount of CsPbBr_3_ NCs hexane solution on glassy carbon electrode (GCE) by drop-casting. The cross-linked surface ligands (OA, OAm) could self-assemble a smooth and robust shell and facilitate superlattice formation, resulting in a strong and stable ECL signal in an aqueous solution.

To fabricate a high-quality CsPbBr_3_ QDs film, Xue et al. (2017) proposed a scraping coating method. In detail, CsPbBr_3_ QDs thick hexane slurry was dropped on GCE and then scraped with a glass rod. Afterward, the modified GCE was dipped into EA for 1 s several times. The established film showed reduced grain size and dense coverage, affording five-fold ECL efficiency in EA (vs. Ru(bpy)_3_^2+^/TPrA) and an ultranarrow fwhm of 24 nm (Figure 2E).

Solution-processed colloidal PeNCs exist flooded long-chain aliphatic ligands on their surface, which reduce the interface conductivity and affect the ECL property. Qiu et al. (2019) investigated the interfacial ECL behavior of CsPbBr_3_ NCs film and proposed a three-phase heterostructure strategy for enhanced ECL intensity. The three-phase interface of GCE/CsPbBr_3_ NCs/acetonitrile was constructed by removing partial NCs film to expose the GCE substrate. They verified that the enhanced ECL intensity is closely related to the effective three-phase interface rather than the exposed area of the GCE surface (Figure 2F).

## Electrochemiluminescence Sensing Application

The ECL sensing application of PeNCs mainly focuses on the quantification of small biological molecules, which show a direct quenching or enhancing effect on the ECL signal. H_2_O_2_ was verified to be involved in the anodic charge transfer of CsPbBr_3_ NCs to produce efficient ECL in an aqueous solution. By using the GCE modified with CsPbBr_3_ NCs, therefore, sensitive H_2_O_2_ sensing could be realized (Huang et al., 2017). Since AA is a powerful coreactant for CsPbBr_3_ NCs, an analogous configuration was also employed for AA detection (Cao et al., 2020b). Because alkaline phosphatase (ALP) can catalyze the production of AA from ascorbic acid 2-phosphate, the AA sensing platform could be further extended for ALP measurement (Wang et al., 2020b). Dopamine could be sensitively detected based on its effective quenching effect on the ECL of CsPbBr_3_ NCs/TPrA (Wang et al., 2020c) or FAPbBr_3_/TPrA (Wang et al., 2020a) system.

Monochromatic ECL of PeNCs illustrates the great potential in building ECL resonance energy transfer (RET) systems. Cao et al. (2020c) synthesized dual-potential ECL emitter of CsPbBr_3_ NCs@HCNS. The anodic ECL of CsPbBr_3_ NCs was quenched by rhodamine 6G due to efficient ECL-RET, while the cathodic ECL of HCNS remained unchanged. Further combining with a DNA probe for CD44 receptors targeting and signal amplification via hybridization chain reaction (HCR), a ratiometric strategy was proposed for the sensitive and accurate evaluation of CD44 expression on MCF-7 cells. Cao et al. (2020a) also designed a 5’-hydroxyl terminal DNA probe, which successively underwent phosphorylation of T4 polynucleotide kinase (T4 PNK), cleavage of *λ*-exonuclease, signal amplification of HCR, and complementary capture of an electrode, a similar ECL-RET system between the self-enhanced CsPbBr_3_-NCDs@HZIF-8 ternary nanocomposite and rhodamine 6G was established for ultrasensitive T4 PNK activity evaluation.

## Conclusion and Outlooks

This mini-review primarily focuses on the state of art of PeNCs in the ECL domain. The topics cover the emerging PeNCs emitters to date, the highlighting on the stabilization and interface manipulation strategies for desirable ECL performance, and a brief summary of the tentative sensing applications. In the past four years, various PeNCs, including fully-inorganic or organometallic lead-based PeNCs, bismuth-based PeNCs, and mixed-ions PeNCs are discovered with ECL activity and their underlying ECL mechanisms are investigated systematically. During the course of experiment, several unique ECL merits are disclosed, such as electrochemically switchable ECL property, ultranarrow ECL linewidth, and composition-tunable ECL performance. In order to meet the challenges in improving the stability and enhancing the charge injection/transfer capacities of PeNCs, elaborate strategies have been proposed, such as surface engineering, nanocomposite construction, and interface manipulation. As presented above, PeNCs are becoming promising alternatives to traditional semiconductor ECL transmitters for idiographic sensing applications.

Despite considerable progress, ECL research on PeNCs is still in its infancy, and huge challenges remain in exploring high-performance PeNCs luminophores and realizing their unique ECL applications. In terms of material synthesis, on the one hand, lead-based PeNCs can be further improved as promising ECL candidates through morphology control and surface engineering. For example, ligands with both co-reaction and stability capabilities can be utilized for surface modification to achieve self-enhanced ECL property. Host-guest assemblies of functional ligands can tailor the highly-passivated NCs surface and unique ECL performance. The correlation between ECL property and the morphology of PeNCs has not been fully disclosed to date. On the other hand, exploring ECL-active lead-free PeNCs with environment friendliness is highly desired. However, their optical properties are still greatly inferior to lead-based counterparts. Thus, developing new synthetic approaches to access highly luminescent and stable lead-free PeNCs is crucial to their ECL progress. The soft ionic crystal structure and high defect tolerance of PeNCs determine the feasibility of ion exchange or doping. Hence, high-quality bimetallic or polymetallic PeNCs can be designed by doping analogous or impurity ions for tunable ECL property. Although the doping of Rb^+^ and Sb^3+^ can boost the ECL intensity of PeNCs, the in-depth mechanism has not been fully elucidated, that is, for example, theoretical computations of energy level arrangement. In addition, the construction of PeNCs-devised nanocomposites is an alternative method, especially in exploring a suitable architecture for the trade-off between ECL efficiency and structural stability.

In terms of ECL applications, the facile PL tunability of PeNCs over the entire visible spectral region through halogen exchange has been universally recognized, but analogous multicolor ECL of PeNCs has not yet been fully achieved because of their relatively low ECL efficiency. The high ECL efficiency and color purity make lead-based PeNCs very attractive for ECL biosensing, but the toxicity issue of Pb^2+^ must be considered, and forming a compact and biocompatible coating might be a good choice. The stability of aqueous ECL remains to be addressed for reproducible analysis results. Nevertheless, it might be a new idea to utilize their instability or crystal conversion to realize some unique sensing applications. Also, their unique ECL properties can be further exploited and utilized, such as electrochemical switchability and monochromaticity, and a deeper understanding of PeNCs-related sensing mechanisms is also indispensable.

## References

[B1] Cai Z. Li F. Xu W. Xia S. Zeng J. He S. (2018). Colloidal CsPbBr_3_ perovskite nanocrystal films as electrochemiluminescence emitters in aqueous solutions. Nano Res. 11, 1447–1455. 10.1007/s12274-017-1760-7

[B2] Cao Y. Zhang Z. Li L. Zhang J.-R. Zhu J. J. (2019). An improved strategy for high-quality cesium bismuth bromine perovskite quantum dots with remarkable electrochemiluminescence activities. Anal. Chem. 91, 8607–8614. 10.1021/acs.analchem.9b01918 31148456

[B3] Cao Y. Zhou Y. Lin Y. Zhu J. J. (2020a). Hierarchical metal-organic framework-confined CsPbBr_3_ quantum dots and aminated carbon dots: a new self-sustaining suprastructure for electrochemiluminescence bioanalsis. Anal. Chem. 93, 1818. 10.1021/acs.analchem.0c04717 33372764

[B4] Cao Y. Zhu W. Li L. Zhang Z. Chen Z. Lin Y. (2020b). Size-selected and surface-passivated CsPbBr_3_ perovskite nanocrystals for self-enhanced electrochemiluminescence in aqueous media. Nanoscale 12, 7321–7329. 10.1039/d0nr00179a 32202287

[B5] Cao Y. Zhu W. Wei H. Ma C. Lin Y. Zhu J. J. (2020c). Stable and monochromatic all-inorganic halide perovskite assisted by hollow carbon nitride nanosphere for ratiometric electrochemiluminescence bioanalysis. Anal. Chem. 92, 4123–4130. 10.1021/acs.analchem.0c00070 32046479

[B6] Chen L. Kang Q. Li Z. Zhang B. Zou G. Shen D. (2020). Tunable electrochemiluminescence properties of CsPbBr_3_ perovskite nanocrystals using mixed-monovalent cations. New J. Chem. 44, 3323–3329. 10.1039/c9nj05665c

[B7] Ding Z. Quinn B. M. Haram S. K. Pell L. E. Korgel B. A. Bard A. J. (2002). Electrochemistry and electrogenerated chemiluminescence from silicon nanocrystal quantum dots. Science 296, 1293–1297. 10.1126/science.1069336 12016309

[B8] Fu K. He Y. Zhang B. Gao X. Zou G. (2020a). Enhanced aqueous stability and radiative-charge-transfer of CsPbBr_3_/Ag_2_S perovskite nanocrystal hybrids. J. Electroanal. Chem. 858, 113835. 10.1016/j.jelechem.2020.113835

[B9] Fu L. Fu K. Hsu H. Y. Gao X. Zou G. (2020b). Ce^4+^ doping to modulate electrochemical and radiative-charge-transfer behaviors of CsPbBr_3_ perovskite nanocrystal. J. Electroanal. Chem. 876, 114546. 10.1016/j.jelechem.2020.114546

[B10] Hao N. Lu J. Dai Z. Qian J. Zhang J. Guo Y. (2019). Analysis of aqueous systems using all-inorganic perovskite CsPbBr_3_ quantum dots with stable electrochemiluminescence performance using a closed bipolar electrode. Electrochem. Commun. 108, 106559. 10.1016/j.elecom.2019.106559

[B11] Huang Y. Fang M. Zou G. Zhang B. Wang H. (2016). Monochromatic and electrochemically switchable electrochemiluminescence of perovskite CsPbBr_3_ nanocrystals. Nanoscale 8, 18734–18739. 10.1039/c6nr06456f 27790659

[B12] Huang Y. Long X. Shen D. Zou G. Zhang B. Wang H. (2017). Hydrogen peroxide involved anodic charge transfer and electrochemiluminescence of all-inorganic halide perovskite CsPbBr_3_ nanocrystals in an aqueous medium. Inorg. Chem. 56, 10135–10138. 10.1021/acs.inorgchem.7b01515 28829127

[B13] Jia J. Fu K. Hou S. Zhang B. Fu L. Hsu H. Y. (2019). Enhanced charge injection and recombination of CsPbBr_3_ perovskite nanocrystals upon internal heterovalent subsitution. J. Phys. Chem. C 123, 29916–29921. 10.1021/acs.jpcc.9b10449

[B14] Kong Y. Zhang B. H. Zeng Z. H. Zhang Y. W. Niu L. (2020). Recent advances in electrochemiluminescence of halide perovskites. Chin. J. Anal. Chem. 48, e20021–e20031. 10.1016/S1872-2040(19)61218-1

[B15] Li L. Zhang Z. Chen Y. Xu Q. Zhang J. R. Chen Z. (2019). Sustainable and self‐enhanced electrochemiluminescent ternary suprastructures derived from CsPbBr_3_ perovskite quantum dots. Adv. Funct. Mater. 29, 1902533. 10.1002/adfm.201902533

[B16] Li Z. Kang Q. Chen L. Zhang B. Zou G. Shen D. (2020). Enhancing aqueous stability and radiative-charge-transfer efficiency of CsPbBr_3_ perovskite nanocrystals via conductive silica gel coating. Electrochim. Acta 330, 135322. 10.1016/j.electacta.2019.135332

[B17] Ma C. Cao Y. Gou X. Zhu J. J. (2020). Recent progress in electrochemiluminescence sensing and imaging. Anal. Chem. 92, 431–454. 10.1021/acs.analchem.9b04947 31679341

[B18] Miao W. (2008). Electrogenerated chemiluminescence and its biorelated applications. Chem. Rev. 108, 2506–2553. 10.1021/cr068083a 18505298

[B19] Peng H. Wu W. Huang Z. Xu L. Sheng Y. Deng H. (2020). Cathodic electrochemiluminescence performance of all-inorganic perovskite CsPbBr_3_ nanocrystals in an aqueous medium. Electrochem. Commun. 111, 106667. 10.1016/j.elecom.2020.106667

[B20] Qiu L. Lin L. Huang Y. Lai Z. Li F. Wang S. (2019). Unveiling the interfacial electrochemiluminescence behavior of lead halide perovskite nanocrystals. Nanoscale Adv. 1, 3957–3962. 10.1039/c9na00456d PMC9417726 36132118

[B21] Stoumpos C. C. Kanatzidis M. G. (2016). Halide perovskites: poor man's high-performance semiconductors. Adv. Mater. 28, 5778–5793. 10.1002/adma.201600265 27174223

[B22] Tan X. Zhang B. Zou G. (2017). Electrochemistry and electrochemiluminescence of organometal halide perovskite nanocrystals in aqueous medium. J. Am. Chem. Soc. 139, 8772–8776. 10.1021/jacs.7b05073 28598607

[B23] Wang X. Y. Wu M. X. Ding S. N. (2020b). Anodic electrochemiluminescence from CsPbBr_3_ perovskite quantum dots for an alkaline phosphatase assay. Chem. Commun. 56, 8099–8102. 10.1039/d0cc03648j 32555912

[B24] Wang X. Yu L. Kang Q. Chen L. Jin Y. Zou G. (2020a). Enhancing electrochemiluminescence of FAPbBr_3_ nanocrystals by using carbon nanotubes and TiO_2_ nanoparticles as conductivity and co-reaction accelerator for dopamine determination. Electrochim. Acta 360, 136992. 10.1016/j.electacta.2020.136992

[B25] Wang Y. Chen T. Huang C. Wang Y. Wu J. Sun B. (2020c). Electrochemically switchable electrochemiluminescent sensor constructed based on inorganic perovskite quantum dots synthesized with microwave irradiation. J. Electroanal. Chem. 867, 114181. 10.1016/j.jelechem.2020.114181

[B26] Wusimanjiang Y. Yadav J. Arau V. Steen A. E. Hammer N. I. Pan S. (2019). Blue electrogenerated chemiluminescence from halide perovskite nanocrystals. J. Anal. Test. 3, 125–133. 10.1007/s41664-018-0082-4

[B27] Xue J. Zhang Z. Zheng F. Xu Q. Xu J. Zou G. (2017). Efficient solid-state electrochemiluminescence from high-quality perovskite quantum dot films. Anal. Chem. 89, 8212–8216. 10.1021/acs.analchem.7b02291 28730817

